# 1187. Analysis of the Clonal Recovery Pattern of *Enterococcus faecium* to Evaluate the Efficacy of Pulsed Xenon Ultraviolet Light (PX-UV) in Bioburden Reduction

**DOI:** 10.1093/ofid/ofac492.1022

**Published:** 2022-12-15

**Authors:** Landon Ashby, Thanuri Navarathna, Piyali Chatterjee, Munok Hwang, Hosoon Choi, Sorabh Dhar, Keith S Kaye, Chetan Jinadatha

**Affiliations:** Central Texas Veterans Health Care System, Temple, Texas; Central Texas Veterans Health Care System, Temple, Texas; Central Texas Veterans Health Care System, Temple, Texas; Central Texas Veterans Health Care System, Temple, Texas; Central Texas Veterans Health Care System, Temple, Texas; Wayne State University, Detroit, Michigan; Rutgers - Robert Wood Johnson Medical School, New Brunswick, New Jersey; Central Texas Veterans Health Care System, Temple, Texas

## Abstract

**Background:**

*Enterococcus faecium (E. faecium)* is a common hospital-associated infection (HAI) that can lead to increased costs, morbidity, and mortality. Pulsed Xenon Ultraviolet light (PX-UV) has been shown to reduce bacterial bioburden levels on surfaces. This study aims to assess the effect of the addition of PX-UV to terminal cleaning on the clonal recovery of *E. faecium* sequence types (STs) using Whole Genome Sequencing (WGS) on patient isolates.

**Methods:**

During 2017 to 2020 a prospective, randomized, double-blinded, sham-controlled, interventional, crossover trial in 2 separate Detroit hospitals (H1 and H2) compared HAI counts after the addition of either PX-UV or a non-UV sham device to terminal cleaning methods. The trial consisted of a total of 16 units randomized to have either the treatment of PX-UV (Group Q) or the sham control device with no UV (Group W) for 12 months. A washout period (Group R) of 6 months followed and the trial concluded with a 12-month crossover of treatments. A total of 60 *E. faecium* samples were collected, then WGS was performed by the Illumina Nextseq 550 instrument. *de novo* assembly was preformed using the SPAdes program. Whole Genome Multilocus Sequence Type (wgMLST) analysis was performed by BioNumerics (v7.6) to construct minimum spanning tree (MST).

**Results:**

A total of 7 STs were obtained across the 2 hospitals (H1 and H2). ST117, ST17, and ST80 all had more than 10 total recovered isolates, ST117 being the most frequent with 24 isolates. Less than 3 isolates were recovered for all other STs. For all STs, Group Q (PX-UV) had 14, Group R (washout period) had 22, and Group W (non-UV sham) had 24. ST18 was only found in Group Q. ST412, ST584, and ST736 were not found in Group W. The data shows that the intervention PX-UV group had a reduction of clonal recovery by 10 STs as compared to the sham UV group.

**Table 1:**
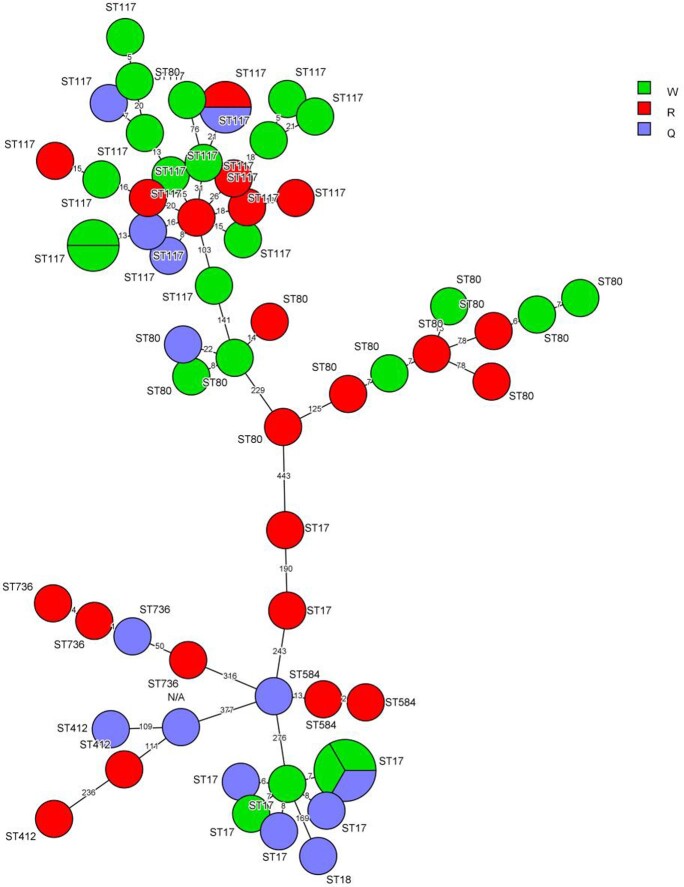
Total number of each sequence type (ST) in H1 and H2

**Figure:**
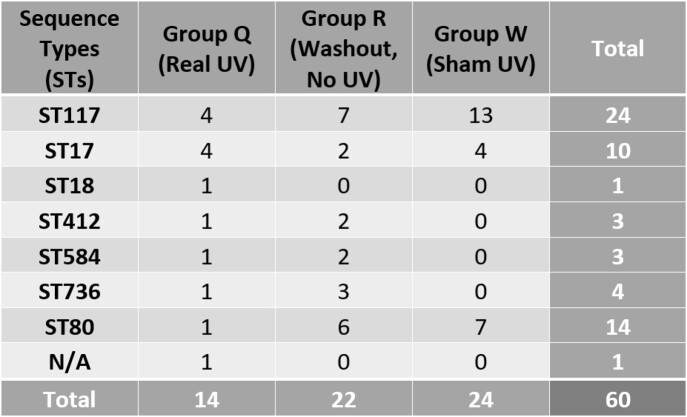

**Conclusion:**

The overall reductions in the number of isolates in the real UV units was driven by reductions in the ST117 a predominant strain in a hospital environment reported previously in Detroit and ST80. There were negligible differences in recovery of other ST between the groups. The reduction in clonal recovery of *E. faecium* isolates in Group Q as compared to Group R might be related to use of PX-UV following terminal room cleaning.

**Disclosures:**

**Piyali Chatterjee, PhD**, AHRQ Grant # 1R03HS027667-01: Grant/Research Support|AHRQ Grant # 1R03HS027667-01: Central Texas Veterans Health Care System **Keith S. Kaye, MD, MPH**, Allecra: Advisor/Consultant|GlaxoSmithKline plc.: Receiving symposia honoraria|GlaxoSmithKline plc.: GlaxoSmithKline plc.-sponsored study 212502|Merck: Advisor/Consultant|qpex: Advisor/Consultant|Shionogi: Grant/Research Support|Spero: Advisor/Consultant **Chetan Jinadatha, MD, MPH**, AHRQ R01 Grant-5R01HS025598: Grant/Research Support|EOS Surfaces: Copper Coupons and materials for testing.

